# Association of Patient Race and Ethnicity With Differences in Opioid Prescribing by Primary Care Physicians for Older Adults With New Low Back Pain

**DOI:** 10.1001/jamahealthforum.2021.2333

**Published:** 2021-09-10

**Authors:** Dan P. Ly

**Affiliations:** 1 Division of General Internal Medicine and Health Services Research, David Geffen School of Medicine, University of California, Los Angeles; 2 VA Greater Los Angeles Healthcare System, Department of Veterans Affairs, Los Angeles, California

## Abstract

**Importance:**

Substantial patient racial and ethnic differences in opioid prescribing have been documented, but how much of these differences were attributable to physicians prescribing opioids differently to patients of racial and ethnic minority groups is unknown, particularly during the first wave of the opioid epidemic when the dangers of opioid prescribing and use were not as well known.

**Objective:**

To examine associations of patient race and ethnicity with differences in opioid prescribing by the same primary care physician (PCP) for new low back pain episodes among older adults from 2007 to 2014.

**Design, Setting, and Participants:**

This cross-sectional study used Medicare data of PCP office visits by Medicare beneficiaries who were 66 years or older with new low back pain.

**Main Outcomes and Measures:**

Prescribing of any opioid in the first year of a new low back pain episode (days 1-365) and subsequent long-term use of an opioid (prescribed for ≥180 days in days 366-730).

**Results:**

Among the study population of 274 771 patients (mean [SD] age, 77.1 [7.2] years; 192 105 [69.9%] women) with new low back pain, 15 285 (6%) were Asian or Pacific Islander, 16 079 (6%) were Black, 21 289 (8%) were Hispanic, and 222 118 (81%) were White, cared for by 63 494 physicians. In adjusted analysis, on average, 11.5% of the White patients (95% CI, 11.4 to 11.6) received an opioid prescription in the first year of new low back pain. The same prescribing physician was 1.5 percentage points (PP; 95% CI, −2.2 PP to −0.8 PP) less likely to prescribe an opioid if the patient was Black, 2.7 PP (95% CI, −3.5 PP to −1.8 PP) less likely if the patient was Asian or Pacific Islander, and 1.0 PP (95% CI, −1.7 PP to −0.3 PP) less likely if the patient was Hispanic. The same physician was more likely to prescribe a prescription nonsteroidal anti-inflammatory drug to a patient of a racial or ethnic minority group. White patients with new low back pain were more likely to develop subsequent long-term opioid use than patients of racial and ethnic minority groups (eg, 1.8% for White patients vs 0.5% for Hispanic patients).

**Conclusions and Relevance:**

This cross-sectional study found that from 2007 to 2014, primary care physicians prescribed opioids for new low back pain more often to White patients than to patients of racial and ethnic minority groups. These results suggest that there may have been unequal treatment of pain by physicians when less was known about the morbidity associated with opioid use.

## Key Points

**Question:**

Were there patient racial and ethnic differences in opioid prescribing by the same primary care physician for episodes of new low back pain, particularly during the first wave of the opioid epidemic when the dangers of opioid use were not as well known?

**Finding:**

In this cross-sectional study of 274 771 older patients with new low back pain, primary care physicians were more likely to prescribe opioids to White patients than to Asian or Pacific Islander, Black, or Hispanic patients.

**Meaning:**

There may have been unequal treatment of pain by physicians when the morbidity associated with opioid use was less well known.

## Teaser

This cross-sectional study uses a large Medicare data set of older adults with low back pain to examine how race and ethnicity may have been associated with opioid prescribing by primary care physicians during the first wave of the opioid epidemic.

## Introduction

Historically, there have been substantial racial and ethnic differences in opioid prescribing practices. These differences have been noted particularly in emergency departments,^1,2,3,4,5^ but they have also been found in the outpatient setting.^6^ These differences have occurred despite research suggesting that patients of racial and ethnic minority groups have the same or higher prevalence and level of pain as White patients.^7,8,9^

Other research^10,11^ has found a 3- to 4-fold variation across physicians in likelihood of prescribing opioids. Prior findings of racial and ethnic differences in opioid prescribing could have been associated with non-Hispanic White patients being more likely to be cared for by physicians who more frequently prescribe opioids. Whether the same physician prescribed opioids less frequently to patients of racial and ethnic minority groups than to White patients has not been explored. Such prescribing differences may suggest differential treatment or bias by physicians, particularly during what the Centers for Disease Control and Prevention calls the first wave (through 2010) of the opioid epidemic when opioid prescribing was rapidly increasing and the dangers of opioid use were not as well known as they are today.^12,13^

The present study used a national sample of older Medicare patients in 2007 to 2014 to examine whether primary care physicians (PCPs), the most frequent prescribers of opioids,^14^ were less likely to prescribe opioids to patients of a racial or ethnic minority group than to White patients for the same outpatient concern, ie, new low back pain.^15,16,17^ This study examined whether physicians were more likely to prescribe a prescription nonsteroidal anti-inflammatory drug (hereafter, NSAID) to patients of a racial or ethnic minority group. This study examined whether such prescribing differences, if they existed, varied depending on the number of visits for back pain during the first year after diagnosis, with the number of visits used as a proxy for the level of or duration of pain. This study examined whether within-physician opioid-prescribing differences changed between 2007 to 2010 and 2011 to 2014. This study also examined whether there were racial and ethnic differences in developing subsequent long-term opioid use.

## Methods

The institutional review board of the National Bureau of Economic Research, where the data were housed and analyzed, approved the study and waived informed consent because only deidentified data were used. This study followed the Strengthening the Reporting of Observational Studies in Epidemiology (STROBE) reporting guideline for cross-sectional studies.

### Data Sources and Study Population

Analyses were performed using 2006 to 2015 claims data for a 20% sample of traditional Medicare beneficiaries, which excludes those enrolled in Medicare Advantage. This study examined episodes of new low back pain, defined as the 365 days after a new diagnosis of low back pain, with no diagnosis code for low back pain and no filled opioid prescription in the prior 365 days.^16,17^ Beneficiaries who were 66 years or older and who were continuously enrolled in Medicare Parts A, B, and D during the year prior to, year of, and year after the diagnosis of new low back pain were included. Only visits where low back pain was 1 of the top 3 diagnoses were included, although this restriction was relaxed in a sensitivity analysis. Definitions of low back pain consistent with prior literature were used (eTable 1 in the Supplement).^18^ Patients with a history of cancer or a hospice claim were excluded. This study focused on 4 racial or ethnic groups: Asian or Pacific Islander, Black, Hispanic, and White non-Hispanic. Race and ethnicity were self-reported, with options defined by the data source. For patients with more than 1 low back pain episode, only the first episode was included.

The receipt of medications was defined using the Part D event file. These data do not include over-the-counter medications, such as nonprescription NSAIDs.

### Outcome Measures

There were 3 outcomes of interest. The first was a binary indicator for opioid prescribing by a particular PCP to a particular patient during an episode of new low back pain (days 1-365). The second was a binary indicator for prescribing NSAIDs by a particular PCP to a particular patient during a new low back pain episode. The third was a binary indicator for subsequent long-term opioid use, which was defined as being prescribed at least 180 days of opioids by all physicians seen in days 366 to 730 (the year after the new low back pain episode). The sample used for the analysis of subsequent long-term opioid use was limited to those who continued not to have cancer or a hospice claim and remained enrolled in Medicare Parts A, B, and D during the examined time period.

### Covariates

In addition to race and ethnicity, patient covariates included age (defined categorically in 5-year age bins), female sex, dual-eligibility for Medicaid, share of months receiving the Part D low-income subsidy, disability as the original reason for Medicare eligibility, Elixhauser comorbidities,^19^ and year of diagnosis of new low back pain.

### Statistical Analysis

In the first specification, a multivariable linear regression of the prescribing of opioids (or NSAIDs) as a function of race and ethnicity was estimated, controlling for the other covariates listed above. Physician fixed effects were included to compare differences in the prescribing of opioids and of NSAIDs to patients of different race and ethnicity by the same physician. The resulting coefficients on race and ethnicity represent the average within-physician opioid prescribing differences by race and ethnicity. Adjusted results by race and ethnicity using marginal standardization (also known as predictive margins) were presented, holding other covariates at their mean values. This study then examined how within-physician racial and ethnic differences in the prescribing of opioids and NSAIDs differed by the number of visits for back pain during a new low back pain episode. To study this, in the second specification, both a categorical variable for number of visits to a particular physician for low back pain (1, 2, 3-4, ≥5 visits) during an episode and the interaction of this variable with the race and ethnicity variable were added. This study then examined whether within-physician opioid-prescribing differences changed between 2007 to 2010 and 2011 to 2014. To study this, in the third specification, the interaction of time period (2011-2014 vs 2007-2010) with race and ethnicity was added. The analysis of the binary outcome of subsequent long-term opioid use also used the first specification. To calculate the percentage of the overall racial and ethnic difference in opioid prescribing for new low back pain attributable to within-physician differences vs between-physician differences, the decomposition method by Chandra and colleagues^20^ was followed (further details in eMethods in the Supplement). Standard errors were clustered at the physician level. All *P* values were from 2-sided tests and results were deemed statistically significant at *P* < .05. For the table of patient characteristics by race and ethnicity, *P* values for differences were calculated using *t* tests for means and χ^2^ tests for categories. All analyses were performed in December 2019 to June 2021 using Stata, version 15.1 (StataCorp LLC).

## Results

Among the study population of 274 771 older patients (mean [SD] age, 77.1 [7.2] years; 192 105 [69.9%] women) with new low back pain, 15 285 (6%) were Asian or Pacific Islander, 16 079 (6%) were Black, 21 289 (8%) were Hispanic, and 222 118 (81%) were White non-Hispanic, cared for by 63 494 physicians (Table 1; eFigure 1 in the Supplement). Women composed 65% of the Asian or Pacific Islander patients in the study sample; 78% of the Black patients; 70% of the Hispanic patients; and 70% of the White patients. Compared with White patients, patients of other races or ethnicities were more likely to be Medicare–Medicaid dual-eligible, have a higher share of months with the Part D low-income subsidy, and have more Elixhauser medical comorbidities. Black and Hispanic patients were more likely to enter Medicare because of disability. In unadjusted analyses, patients of a racial or ethnic minority group were less likely to be prescribed an opioid and more likely to be prescribed an NSAID for an episode of new low back.

**Table 1.** Patient Characteristics by Race and Ethnicity in a Sample Population of Older Adults With a New Episode of Low Back Pain, 2007 to 2014

Characteristic	Race and ethnicity, No. (%)				*P* value for difference between groups		
	Asian or Pacific Islander	Black	Hispanic	White, non-Hispanic	Asian or Pacific Islander and White	Black and White	Hispanic and White
Total	15 285 (6)	16 079 (6)	21 289 (8)	222 118 (81)	NA	NA	NA
Mean age, y	77.1	76.4	76.5	77.3	.02	<.001	<.001
Age group, y							
66-69	2528 (16.5)	3552 (22.1)	4213 (19.8)	41 593 (18.7)		<.001	
70-74	4182 (27.4)	4432 (27.6)	5971 (28.0)	57 824 (26.0)			
75-79	3666 (24.0)	3480 (21.6)	4945 (23.2)	46 599 (21.0)			
80-84	2777 (18.2)	2378 (14.8)	3517 (16.5)	38 775 (17.5)			
85-89	1462 (9.6)	1440 (9.0)	1840 (8.6)	25 052 (11.3)			
90-94	536 (3.5)	592 (3.7)	648 (3.0)	9992 (4.5)			
≥95	134 (0.9)	205 (1.3)	155 (0.7)	2283 (1.0)			
Men	5382 (35.2)	3546 (22.1)	6361 (29.9)	67 377 (30.3)	<.001	<.001	.17
Women	9903 (64.8)	12 533 (77.9)	14 928 (70.1)	154 741 (69.7)	<.001	<.001	.17
Medicare–Medicaid dual eligible	11 918 (78.0)	8522 (53.0)	15 718 (73.8)	34 066 (15.3)	<.001	<.001	<.001
Mean share of months with low-income subsidy (%)	79.6	60.9	78.1	18.0	<.001	<.001	<.001
Originally disabled	488 (3.2)	3306 (20.6)	2785 (13.1)	17 162 (7.7)	<.001	<.001	<.001
Mean Elixhauser score^a^	3.15	3.56	3.86	2.98	<.001	<.001	<.001
Elixhauser comorbidity							
Congestive heart failure	1408 (9.2)	2434 (15.1)	3069 (14.4)	24 249 (10.9)	<.001	<.001	<.001
Arrhythmia	2498 (16.3)	2917 (18.1)	3929 (18.5)	50 111 (22.6)	<.001	<.001	<.001
Valvular disease	2076 (13.6)	2011 (12.5)	3241 (15.2)	30 972 (13.9)	.21	<.001	<.001
Pulmonary circulation disorder	198 (1.3)	551 (3.4)	436 (2.0)	6003 (2.7)	<.001	<.001	<.001
Peripheral vascular disorder	2185 (14.3)	3226 (20.1)	5360 (25.2)	37 162 (16.7)	<.001	<.001	<.001
Uncomplicated hypertension	12 056 (78.9)	14 309 (89.0)	17 577 (82.6)	165 870 (74.7)	<.001	<.001	<.001
Paralysis	154 (1.0)	133 (0.8)	180 (0.8)	1458 (0.7)	<.001	.01	.001
Other neurological disorder	614 (4.0)	856 (5.3)	1308 (6.1)	11 854 (5.3)	<.001	.94	<.001
Chronic pulmonary disease	3197 (20.9)	3354 (20.9)	5497 (25.8)	45 992 (20.7)	.54	.64	<.001
Uncomplicated diabetes	6180 (40.4)	6954 (43.2)	10 057 (47.2)	56 237 (25.3)	<.001	<.001	<.001
Complicated diabetes	1913 (12.5)	2824 (17.6)	4141 (19.5)	17 646 (7.9)	<.001	<.001	<.001
Hypothyroidism	2805 (18.4)	2598 (16.2)	5319 (25.0)	52 623 (23.7)	<.001	<.001	<.001
Kidney failure	1487 (9.7)	2183 (13.6)	2211 (10.4)	17 599 (7.9)	<.001	<.001	<.001
Liver disease	1568 (10.3)	589 (3.7)	1389 (6.5)	6713 (3.0)	<.001	<.001	<.001
Peptic ulcer disease	775 (5.1)	246 (1.5)	452 (2.1)	2607 (1.2)	<.001	<.001	<.001
AIDS/HIV	6 (0.04)	33 (0.2)	28 (0.1)	54 (0.02)	.26	<.001	<.001
Rheumatoid arthritis	908 (5.9)	1062 (6.6)	2115 (9.9)	16 991 (7.6)	<.001	<.001	<.001
Coagulopathy	317 (2.1)	358 (2.2)	718 (3.4)	6000 (2.7)	<.001	<.001	<.001
Obesity	261 (1.7)	1338 (8.3)	1604 (7.5)	10 281 (4.6)	<.001	<.001	<.001
Weight loss	832 (5.4)	1016 (6.3)	863 (4.1)	8762 (3.9)	<.001	<.001	.44
Electrolyte disorder	1594 (10.4)	1866 (11.6)	2216 (10.4)	22 236 (10.0)	.10	<.001	.06
Blood loss anemia	209 (1.4)	241 (1.5)	280 (1.3)	2593 (1.2)	.03	<.001	.06
Deficiency anemia	1312 (8.6)	1679 (10.4)	2749 (12.9)	17 317 (7.8)	<.001	<.001	<.001
Alcohol abuse	42 (0.3)	88 (0.5)	121 (0.6)	888 (0.4)	.02	.005	<.001
Drug abuse	23 (0.2)	50 (0.3)	70 (0.3)	513 (0.2)	.04	.04	.005
Psychoses	142 (0.9)	488 (3.0)	481 (2.3)	4507 (2.0)	<.001	<.001	.02
Depression	833 (5.4)	1166 (7.3)	3196 (15.0)	25 054 (11.3)	<.001	<.001	<.001
Complicated hypertension	2564 (16.8)	2594 (16.1)	3579 (16.8)	18 744 (8.4)	<.001	<.001	<.001
Mean No. of visits during a new episode	1.75	1.53	1.64	1.53	<.001	.84	<.001
No. of visits							
1	9914 (64.9)	11 575 (72.0)	14 544 (68.3)	159 314 (71.7)		<0.001	
2	3162 (20.7)	2875 (17.9)	4177 (19.6)	40 620 (18.3)			
3-4	1689 (11.1)	1300 (8.1)	1969 (9.2)	18 334 (8.3)			
≥5	520 (3.4)	329 (2.0)	599 (2.8)	3850 (1.7)			
Opioid prescribed	966 (6.3)	1752 (10.9)	1961 (9.2)	26 801 (12.1)	<.001	<.001	<.001
NSAID prescribed	5938 (38.8)	4576 (28.5)	7900 (37.1)	50 508 (22.7)	<.001	<.001	<.001

^a^ Elixhauser comorbidity software identifies up to 28 noncancer comorbidities, such as hypertension and diabetes, based on diagnosis codes found in the administrative data, and the Elixhauser score is the sum of these comorbidities.

Abbreviations: NA, not applicable; NSAID, nonsteroidal anti-inflammatory drug.

**Figure 1.**
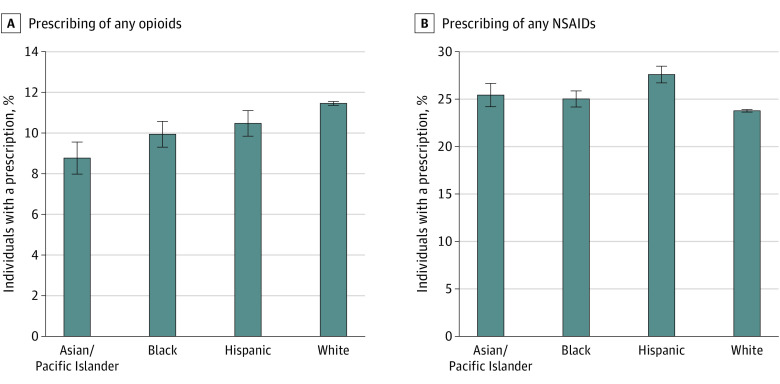
Racial and Ethnic Differences in Prescribing Opioids and NSAIDs by the Same Physician for New Episodes of Low Back Pain, 2007 to 2014 Error bars represent 95% CIs, and NSAIDs denotes nonsteroidal anti-inflammatory drugs.

In adjusted analyses, a physician was less likely to prescribe opioids to patients of a racial or ethnic minority group than to White patients during an episode of new low back pain (Figure 1A; eTable 2 in the Supplement). On average, physicians prescribed opioids to 11.5% (95% CI, 11.4% to 11.6%) of White patients; 9.9% (95% CI, 9.3% to 10.6%) of Black patients, a difference of 1.5 percentage points (PP; 95% CI, −2.2 PP to −0.8 PP) compared with White patients; 8.8% (95% CI, 8.0% to 9.6%) of Asian or Pacific Islander patients, a difference of 2.7 PP (95% CI, −3.5 PP to −1.8 PP); and 10.5% (95% CI, 9.8% to 11.1%) of Hispanic patients, a difference of 1.0 PP (95% CI, −1.7 PP to −0.3 PP). These results were largely unchanged when relaxing the restriction that low back pain must be 1 of the top 3 diagnoses (eTable 3 in the Supplement) and when using a logistic rather than a linear model (eTable 4 in the Supplement). Results were unchanged when controlling for state or zip code (results not shown). A physician was more likely to prescribe an opioid to a White patient than to a Black patient and to an Asian or Pacific Islander patient within 1 month, 2 months, and 3 months of the date of the initial diagnosis of low back pain (eTable 5 in the Supplement).

Conversely, on average, a physician prescribed NSAIDs to 23.8% (95% CI, 23.6% to 23.9%) of White patients, to 25.0% (95% CI, 24.2% to 25.9%) of Black patients, a difference of 1.2 PP (95% CI, 0.3 PP to 2.2 PP); 25.4% (95% CI, 24.2% to 26.6%) of Asian or Pacific Islander patients, a difference of 1.6 PP (95% CI, 0.3 PP to 3.0 PP); and 27.6% (95% CI, 26.7% to 28.5%) of Hispanic patients, a difference of 3.8 PP (95% CI, 2.9 PP to 4.8 PP) (Figure 1B; eTable 2 in the Supplement). Results for both subsequent opioid prescribing and subsequent NSAID prescribing were substantively unchanged when excluding those with any NSAID use within 30 days or within 365 days before the diagnosis of new low back pain (eTable 6 in the Supplement).

**Figure 2.**
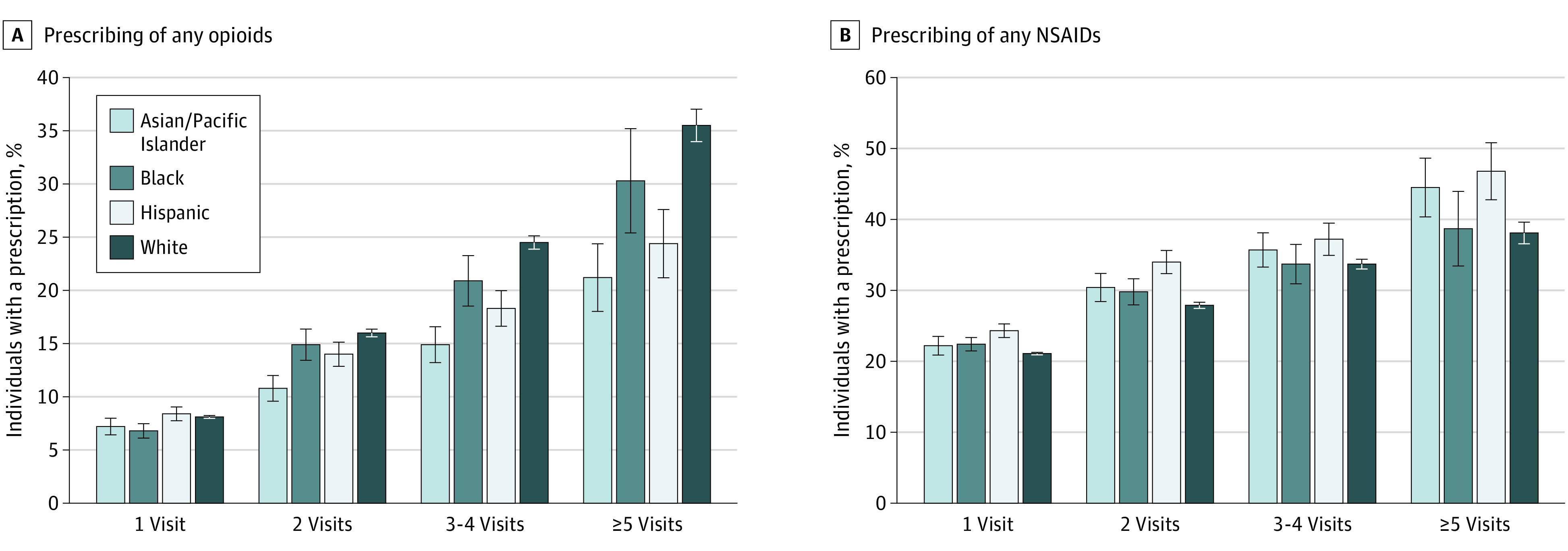
Racial and Ethnic Differences in Prescribing Opioids and NSAIDs by the Same Physician for New Episodes of Low Back Pain by Number of Visits for Low Back Pain, 2007 to 2014 Error bars represent 95% CIs, and NSAIDs denotes on steroidal anti-inflammatory drugs.

The probability of prescribing opioids and NSAIDs increased with the number of visits for back pain during a new low back pain episode (Figure 2A and B). In adjusted analyses, on average, physicians prescribed opioids to 35.5% (95% CI, 34.0% to 37.0%) of White patients with 5 or more visits for back pain during a back pain episode, while they were 5.2 PP (95% CI, −10.3 PP to −0.09 PP) less likely to prescribe opioids to Black patients with 5 or more visits, 14.3 PP (95% CI, −17.8 PP to −10.7 PP) less likely to prescribe opioids to Asian or Pacific Islander patients, and 11.1 PP (95% CI, −14.7 PP to −7.6 PP) less likely to prescribe opioids to Hispanic patients (Figure 2A). Meanwhile, on average, physicians prescribed NSAIDs to 38.1% (95% CI, 36.6% to 39.7%) of White patients with 5 or more visits for back pain during a back pain episode, while they were 6.3 PP (95% CI, 1.9 PP to 10.7 PP) more likely to prescribe NSAIDs to Asian or Pacific Islander patients and 8.6 PP (95% CI, 4.3 PP to 12.9 PP) more likely to prescribe NSAIDs to Hispanic patients (Figure 2B).

**Figure 3.**
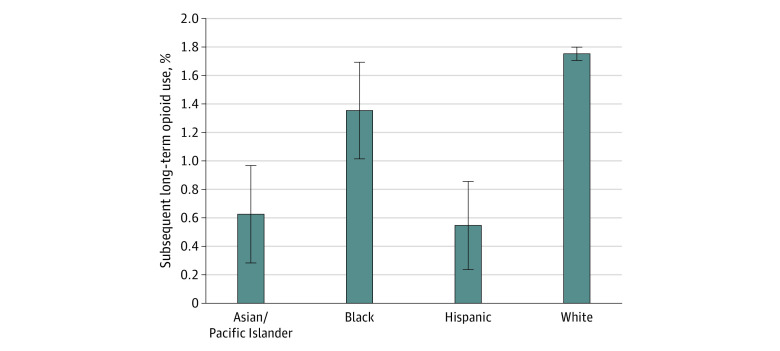
Racial and Ethnic Differences in Subsequent Long-term Opioid Use After New Episode of Low Back Pain, 2007 to 2014 Error bars represent 95% CIs.

Although the change in the likelihood of prescribing opioids for new low back pain episodes decreased by 1.0 PP (95% CI, −1.3 PP to −0.7 PP) for White patients between 2007 to 2010 and 2011 to 2014, this study did not find a decrease that was statistically significantly different from the decrease among patients of a racial or ethnic minority group (Table 2). In adjusted analyses (Figure 3), long-term opioid use developed during the year after the new low back pain episode in 1.8% of White patients (95% CI, 1.7% to 1.8%); 1.4% of Black patients (95% CI, 1.0% to 1.7%), a difference of 0.4 PP (95% CI, −0.8 PP to −0.03 PP); 0.6% of Asian or Pacific Islander patients (95% CI, 0.3% to 1.0%), a difference of 1.1 PP (95% CI, −1.5 PP to −0.8 PP); and 0.5% of Hispanic patients (95% CI, 0.2% to 0.9%), a difference of 1.2 PP (95% CI, −1.5 PP to −0.9 PP). When decomposing the overall racial and ethnic difference in opioid prescribing between patients of racial or ethnic minority groups and White patients to differences across prescribing physicians and to within-physician differences, only 40% of the opioid prescribing difference was attributable to differences across prescribing physicians, meaning that 60% of the racial and ethnic difference in opioid prescribing for new low back pain remained, even when comparing patients seen by the same physician (eTable 7 in the Supplement).

**Table 2.** Trend in Racial and Ethnic Differences in Opioids Prescribed by the Same Physician for New Episodes of Low Back Pain, 2007 to 2014

Race and ethnicity	% (95% CI)		Difference, percentage points (95% CI)
	2007-2010	2011-2014	
Asian or Pacific Islander	9.1 (8.2 to 10.0)	8.4 (7.4 to 9.3)	−0.7 (−1.6 to 0.2)
Black	10.2 (9.3 to 11.0)	9.6 (8.7 to 10.4)	−0.6 (−1.7 to 0.5)
Hispanic	10.7 (10.0 to 11.4)	10.2 (9.3 to 11.0)	−0.6 (−1.5 to 0.4)
White	11.9 (11.7 to 12.1)	10.9 (10.7 to 11.1)	−1.0 (−1.3 to −0.7)

## Discussion

Using a nationally representative data set of older Medicare patients, this cross-sectional study found that the same PCP was less likely to prescribe an opioid and more likely to prescribe an NSAID for new low back pain to patients of a racial or ethnic minority group than to White patients. These prescribing differences were larger for patients with more visits for back pain during a given back pain episode. This study failed to find a narrowing of these prescribing differences between 2007 to 2010 and 2011 to 2014. In the year after a new low back pain episode, White patients were more likely than patients of a racial or ethnic minority group to develop subsequent long-term opioid use. Of the overall racial and ethnic difference in opioid prescribing for new low back pain, 60% was attributable to within-physician differences.

To our knowledge, no other study has examined racial and ethnic differences in opioid prescribing by the same physician. Prior findings of differences by race and ethnicity in opioid prescribing^1,2,3,4,5,6^ encompass both differences across physicians (some may receive an opioid prescription because they were cared for by a higher opioid-prescribing physician) and differences within physicians. By including physician fixed effects, this study controlled for differential patient sorting by race and ethnicity across physicians. Prior studies may not have been able to do this because commonly used data sets to study opioid prescribing, particularly the National Hospital Ambulatory Medical Care Survey^1,3,4,5^ and the National Ambulatory Medical Care Survey,^6^ randomly sample visits from a 4-week and 1-week reporting period, respectively.^21^ Therefore, studies using these data sets do not have large enough sample sizes per physician to examine prescribing differences by the same physician. In addition, this study found that differential sorting of patients accounted for less than half of the overall racial and ethnic difference in opioid prescribing, meaning that more than half of this difference was attributable to the same physician prescribing opioids differently to patients of a racial or ethnic minority group compared with White patients.

A possible reason for the observed differences in opioid prescribing by the same physician is that patients of a racial or ethnic minority group ask for opioid medications less frequently than White patients do. However, research finds that patients of a racial or ethnic minority group have similar expectations of pain relief as White patients^22^ and the same or a higher prevalence of and level of pain as White patients.^7,8,9^ While the present study’s findings do not exclude the possibility that racial and ethnic differences in preferences for opioid use may drive some of the observed results, if a greater number of visits for low back pain provides a clearer signal to physicians about the severity or persistence of the pain, prescribing differences would be expected to narrow, not increase, with a higher number of visits.

A more worrying possibility for these differences is that physicians may have different levels of trust in patients by race and ethnicity. A survey^23^ found that PCPs had lower trust (less confidence that the patient would “not manipulate the office visit for secondary gain [eg, …prescription of controlled substances]”) in non-White than in White patients. Physicians in that study had differences in their level of trust despite there being no racial or ethnic difference in opioid misuse in patients.^23^ In fact, other research^24^ suggests that White patients were more likely than patients of other races and ethnicities to have an opioid use disorder.

The time period examined in this study includes the first wave of the opioid epidemic when opioid prescribing was rapidly increasing^12,13^ and the morbidity associated with opioids was less known than it is today. For example, in 2011, the Institute of Medicine published the report *Relieving Pain in America,* in which they wrote that “the majority of people with pain use their prescription drugs properly, are not a source of misuse, and should not be stigmatized or denied access because of the misdeeds or carelessness of others.”^25^ In light of this sentiment among physicians during this time period—that opioids were safe for most people and that only a few patients might misuse opioids—the prescribing patterns found in this study suggest differential treatment or even bias by physicians when managing patient pain. Finally, although this study describes opioid prescribing differences by race and ethnicity for the same physician, the opioid epidemic has had deleterious effects for people of all races and ethnicities.^26^ Public policy responses that address current racial and ethnic disparities will be important,^27,28,29^ such as making medication-assisted treatment (eg, prescribing buprenorphine) more accessible by offering incentives to physicians who serve patients of racial and ethnic minority groups and populations with low income.

### Limitations

This study had several limitations. First, this was an observational study and residual confounding is possible. Second, results were limited to the traditional Medicare population with Medicare Part D and may not generalize to other populations, including younger populations, those without Medicare Part D, and those with Medicare Advantage. Those with Medicare Advantage represent one-third of all Medicare beneficiaries and historically have been healthier,^30^ so results that included those with Medicare Advantage may have been quite different. Third, results were based on health claims data only; therefore, details of the case history, such as pain intensity and findings of the physical examination, were not included. Fourth, over-the-counter medications are not included in the data, so the use of these medications (eg, acetaminophen and ibuprofen) could not be examined. Fifth, although this study focused on an opioid-naive population, opioid prescriptions were not tied to diagnoses, so they may have been prescribed for other reasons. However, differences were also found when examining opioid prescribing within a few months of the new low back pain diagnosis and when examining opioid prescribing for patients with a large number of visits for low back pain. Sixth, the race and ethnicity variable used does not completely identify patients of racial and ethnic minority groups, especially beneficiaries who are Asian or Pacific Islander or Hispanic^31^; this misidentification would bias the observed differences toward zero. Seventh, the definition of new low back pain used in this study was not externally validated.

## Conclusions

This cross-sectional study found that in a large national sample of older adults with Medicare, physicians prescribed opioids less frequently to patients of racial and ethnic minority groups than to White patients during and after the first wave of the opioid epidemic when opioid prescribing was rapidly increasing. Reasons for these differences and implications of these differences on opioid-related morbidity and mortality should be further explored.

## Notes

Supplement.
eMethods
**eFigure 1.** Flow Diagram for Patients **eTable 1.** List of ICD-9 Codes for Low Back Pain **eTable 2.** Racial and Ethnic Differences within Physician in Opioid Prescribing and in Prescription NSAID Prescribing for Patients with New Low Back Pain, 2007-2014 **eTable 3.** Racial and Ethnic Differences within Physician in Opioid Prescribing for Patients with New Low Back Pain (no restriction to low back pain as one of top 3 diagnoses), 2007-2014 **eTable 4.** Racial and Ethnic Differences within Physician in Opioid Prescribing for Patients with New Low Back Pain (logistic model), 2007-2014 **eTable 5.** Racial and Ethnic Differences within Physician in Timing of Opioid Prescribing for Patients with New Low Back Pain, 2007-2014 **eTable 6.** Racial and Ethnic Differences within Physician in Opioid Prescribing and in Prescription NSAID Prescribing for Patients with New Low Back Pain, 2007-2014 (excluding those with any prescription NSAID use within 30 days or within 365 days prior to new low back pain diagnosis) **eTable 7.** Percent of Overall Racial and Ethnic Difference in Opioid Prescribing for Patients with New Low Back Pain Attributable to Within-Physician Differences, 2007-2014
