# Supplementary material for: Association of Patient Race and Ethnicity With Differences in Opioid Prescribing by Primary Care Physicians for Older Adults With New Low Back Pain
Source: JAMA Health Forum. 2021 Sep 10;2(9):e212333. doi: 10.1001/jamahealthforum.2021.2333 (PMC8796941; doi:10.1001/jamahealthforum.2021.2333)
Supplement: Supplement. — eMethods eFigure 1. Flow Diagram for Patients eTable 1. List of ICD-9 Codes for Low Back Pain eTable 2. Racial and Ethnic Differences within Physician in Opioid Prescribing and in Prescription NSAID Prescribing for Patients with New Low Back Pain, 2007-2014 eTable 3. Racial and Ethnic Differences within Physician in Opioid Prescribing for Patients with New Low Back Pain (no restriction to low back pain as one of top 3 diagnoses), 2007-2014 eTable 4. Racial and Ethnic Differences within Physician in Opioid Prescribing for Patients with New Low Back Pain (logistic model), 2007-2014 eTable 5. Racial and Ethnic Differences within Physician in Timing of Opioid Prescribing for Patients with New Low Back Pain, 2007-2014 eTable 6. Racial and Ethnic Differences within Physician in Opioid Prescribing and in Prescription NSAID Prescribing for Patients with New Low Back Pain, 2007-2014 (excluding those with any prescription NSAID use within 30 days or within 365 days prior to new low back pain diagnosis) eTable 7. Percent of Overall Racial and Ethnic Difference in Opioid Prescribing for Patients with New Low Back Pain Attributable to Within-Physician Differences, 2007-2014 [file jamahealthforum-e212333-s001.pdf]

Supplemental Online Content

Ly DP. Association of patient race and ethnicity with differences in opioid prescribing by primary care physicians for older adults with new low back pain. *JAMA Health Forum*. 2021;2(9): e212333. doi:10.1001/jamahealthforum.2021.2333

eMethods

eFigure 1: Flow Diagram for Patients

eTable 1: List of ICD-9 Codes for Low Back Pain

eTable 2: Racial and Ethnic Differences within Physician in Opioid Prescribing and in Prescription NSAID Prescribing for Patients with New Low Back Pain, 2007-2014

eTable 3: Racial and Ethnic Differences within Physician in Opioid Prescribing for Patients with New Low Back Pain (no restriction to low back pain as one of top 3 diagnoses), 2007-2014

eTable 4: Racial and Ethnic Differences within Physician in Opioid Prescribing for Patients with New Low Back Pain (logistic model), 2007-2014

eTable 5: Racial and Ethnic Differences within Physician in Timing of Opioid Prescribing for Patients with New Low Back Pain, 2007-2014

eTable 6: Racial and Ethnic Differences within Physician in Opioid Prescribing and in Prescription NSAID Prescribing for Patients with New Low Back Pain, 2007-2014 (excluding those with any prescription NSAID use within 30 days or within 365 days prior to new low back pain diagnosis)

eTable 7: Percent of Overall Racial and Ethnic Difference in Opioid Prescribing for Patients with New Low Back Pain Attributable to Within-Physician Differences, 2007-2014

This supplemental material has been provided by the authors to give readers additional information about their work.

© 2021 Ly DP. *JAMA Health Forum.*

eMethods

The analytic file was derived from the following data sets:

1) Outpatient visits and diagnoses were drawn from the Medicare Carrier File

2) Beneficiary enrollment and demographic characteristics were drawn from Medicare Beneficiary Summary File

3) Prescriptions were drawn from the Part D Drug Event File

The Part D Drug Event File includes beneficiary, prescriber, National Drug Code (NDC), branded name, generic name, strength, quantity dispensed, and days supplied. More details can be found here (https://www.resdac.org/cms-data/files/pde/data-documentation).

Physician specialty is contained in the Medicare Carrier File (https://resdac.org/cms-data/variables/line-cms-provider-specialty-code). A primary care physician was a priori defined as those practicing in the following specialties: 1- general practice; 8-family practice; 11-internal medicine; 38-geriatric medicine; 84-preventive medicine.

Race and ethnicity information in the Medicare data was originally collected by the Social Security Administration at the time of Social Security Number application and an imputation algorithm was created by the Research Triangle Institute to improve the sensitivity in identifying minority beneficiaries (Filice CE, Joynt KE. Examining race and ethnicity information in Medicare administrative data. Med Care. 2017; 55: e170-76).

I decompose the difference in prescription of any opioid between White patients and minority patients as follows:

$Opioid^{minority}-Opioid^{White}$

$=\sum\theta_p^{minority}*Opioid_p^{minority}-\sum\theta_p^{White}*Opioid_p^{White}$

$=\sum\theta_p^{White}*(Opioid_p^{minority}-Opioid_p^{White})+\sum(\theta_p^{minority}-\theta_p^{White})*Opioid_p^{minority}$

=within-physician difference + between-physician difference

Where $Opioid_p^{minority}$ and $Opioid_p^{White}$ are risk-adjusted rate of prescription of any opioid for minority and White patients seeing physician *p* and $\theta_p^{minority}$ and θ_p^{White} are the national share of minority and White patients for physician *p*.

The "within-physician difference" term refers to the difference in opioid prescribing by the same physician between their minority patients and their White patients. The "between-physician difference" term refers to the difference in opioid prescribing due to differences in physicians seen between minority patients and White patients, or in other words, minority patients seeing different physicians who prescribe opioids differently than White patients.

© 2021 Ly DP. *JAMA Health Forum.*

eFigure 1: Flow Diagram for Patients

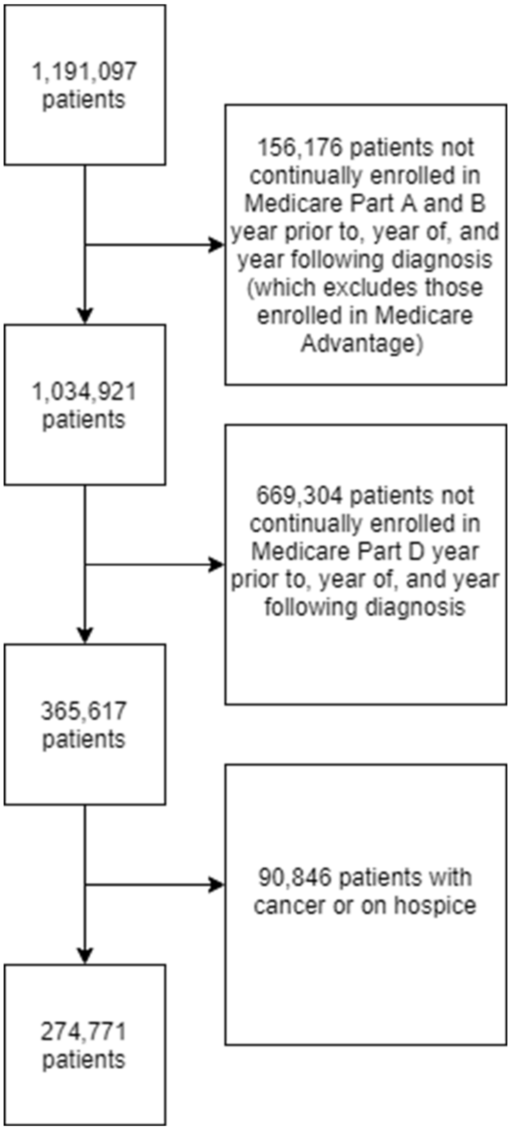

© 2021 Ly DP. *JAMA Health Forum.*

eTable 1: List of ICD-9 Codes for Low Back Pain

7213x, 72190, 72210, 72252, 7226x, 72293, 72402, 7242x, 7243x, 7245x, 7246x, 72470, 72471, 72479, 7385x, 7393x, 7394x, 846xx, 8472x

Note: These ICD-9 codes were crosswalked to ICD-10 codes using a CMS-derived crosswalk found here (https://www.nber.org/data/icd9-icd-10-cm-and-pcs-crosswalk-general-equivalence-mapping.html).

© 2021 Ly DP. *JAMA Health Forum.*

eTable 2: Racial and Ethnic Differences within Physician in Opioid Prescribing and in Prescription NSAID Prescribing for Patients with New Low Back Pain, 2007-2014

	Prescribing of any opioid during a given episode (percentage points)	Prescribing of any prescription NSAID during a given episode (percentage points)
Race and ethnicity		
White, non-Hispanic	-	-
Black	-1.5 (-2.2 to -0.8)	1.2 (0.3 to 2.2)
Asian/Pacific Islander	-2.7 (-3.5 to -1.8)	1.6 (0.3 to 3.0)
Hispanic	-1.0 (-1.7 to -0.3)	3.8 (2.9 to 4.8)
Age		
66-69	-	-
70-74	0.2 (-0.2 to 0.5)	-1.1 (-1.6 to -0.6)
75-79	0.2 (-0.2 to 0.6)	-2.3 (-2.9 to -1.8)
80-84	0.4 (-0.06 to 0.8)	-3.4 (-4.0 to -2.9)
85-89	0.2 (-0.3 to 0.7)	-5.0 (-5.6 to -4.3)
90-94	0.3 (-0.4 to 1.1)	-6.2 (-7.1 to -5.3)
95+	-0.1 (-1.5 to 1.2)	-8.8 (-10.4 to -7.2)
Sex		
Male	-	-
Female	-0.1 (-0.4 to 0.2)	0.8 (0.4 to 1.2)
Medicare-Medicaid dual eligible	0.04 (-0.6 to 0.7)	3.0 (2.1 to 3.9)
Share of months with low-income subsidy	1.0 (0.4 to 1.7)	2.1 (1.3 to 2.9)
Originally disabled	1.8 (1.3 to 2.4)	-0.5 (-1.1 to 0.2)
Elixhauser co-morbidities		
Congestive heart failure	1.7 (1.2 to 2.2)	-1.8 (-2.4 to -1.2)
Arrhythmia	0.9 (0.6 to 1.3)	-3.9 (-4.4 to -3.5)
Valvular disease	-0.002 (-0.4 to 0.4)	-1.0 (-1.6 to -0.5)

© 2021 Ly DP. *JAMA Health Forum.*

Pulmonary circulation disorder	1.7 (0.7 to 2.6)	-1.7 (-2.7 to -0.6)
Peripheral vascular disorder	1.1 (0.7 to 1.5)	-0.8 (-1.2 to -0.3)
Uncomplicated hypertension	1.5 (1.1 to 1.8)	1.2 (0.8 to 1.7)
Paralysis	1.7 (-0.03 to 3.3)	-2.5 (-4.4 to -0.5)
Other neurological disorder	0.8 (0.2 to 1.4)	-1.0 (-1.8 to -0.3)
Chronic pulmonary disease	2.0 (1.7 to 2.3)	-0.3 (-0.7 to 0.1)
Uncomplicated diabetes	1.2 (0.9 to 1.5)	0.3 (-0.2 to 0.7)
Complicated diabetes	1.0 (0.4 to 1.5)	-0.7 (-1.4 to -0.05)
Hypothyroidism	-0.1 (-0.4 to 0.2)	0.2 (-0.2 to 0.6)
Renal failure	1.2 (0.6 to 1.7)	-5.7 (-6.3 to -5.1)
Liver disease	0.5 (-0.2 to 1.2)	-1.9 (-2.8 to -1.0)
Peptic ulcer disease	2.1 (0.9 to 3.2)	0.5 (-1.0 to 2.0)
AIDS/HIV	0.9 (-5.7 to 7.4)	-1.2 (-9.4 to 7.0)
Rheumatoid arthritis	4.2 (3.6 to 4.7)	2.2 (1.5 to 2.8)
Coagulopathy	2.0 (1.1 to 2.8)	-2.3 (-3.3 to -1.3)
Obesity	1.3 (0.6 to 1.9)	1.6 (0.8 to 2.4)
Weight loss	2.0 (1.3 to 2.7)	-2.3 (-3.1 to -1.5)
Electrolyte disorder	2.5 (2.0 to 3.0)	-0.5 (-1.1 to 0.07)
Blood loss anemia	0.2 (-1.0 to 1.5)	-0.9 (-2.5 to 0.6)
Deficiency anemia	0.7 (0.2 to 1.2)	-0.3 (-0.9 to 0.4)
Alcohol abuse	2.1 (-0.2 to 4.3)	-1.9 (-4.4 to 0.7)
Drug abuse	5.0 (2.0 to 8.1)	-2.9 (-6.1 to 0.4)
Psychoses	-0.4 (-1.4 to 0.6)	0.02 (-1.2 to 1.2)
Depression	2.3 (1.8 to 2.7)	0.9 (0.4 to 1.5)
Complicated hypertension	0.4 (-0.1 to 0.9)	-0.5 (-1.2 to 0.1)

Notes: Author's calculation using Medicare data from 2006 to 2015. The examined time period is 2007-2014; 2006 data are used to look for a diagnosis code for low back pain or for a filled opioid prescription in the 365 days prior to a low back pain diagnosis in 2007, while 2015 data are used to look for filled opioid or NSAID prescriptions in the 365 days following a new diagnosis of low back pain in 2014. Multivariable regression (linear probability model) of opioid prescribing and of prescription NSAID prescribing as a function of race and ethnicity were performed; physician fixed effects were included to compare differences in the prescribing of opioids to patients of different race and ethnicity within the same physician, controlling also for the other covariates listed above (year was also controlled for, but the coefficients on year are not reported). For race and ethnicity, White non-Hispanic is the reference category. For age, patients aged 66-69 are the reference category. For sex, men are the reference category. Using marginal standardization, the adjusted value for White was 11.5 (95% CI 11.4 to 11.6), the adjusted value for age 66-69 was 11.0 (95% CI 10.7 to 11.2), and the adjusted value for male was 11.2 (95% CI 11.0 to 11.4). Standard errors were clustered at the physician level. 95% confidence interval in parentheses.

© 2021 Ly DP. *JAMA Health Forum.*

eTable 3: Racial and Ethnic Differences within Physician in Opioid Prescribing for Patients with New Low Back Pain (no restriction to low back pain as one of top 3 diagnoses), 2007-2014

	Prescribing of any opioid during a given episode (percentage points)
Race and ethnicity	
White, non-Hispanic	-
Black	-1.3 (-1.9 to -0.7)
Asian/Pacific Islander	-2.4 (-3.1 to -1.6)
Hispanic	-0.8 (-1.5 to -0.2)

Notes: Author's calculation using Medicare data from 2006 to 2015. The examined time period is 2007-2014; 2006 data are used to look for a diagnosis code for low back pain or for a filled opioid prescription in the 365 days prior to a low back pain diagnosis in 2007, while 2015 data are used to look for filled opioid or NSAID prescriptions in the 365 days following a new diagnosis of low back pain in 2014. The multivariable linear regression underlying these results included age (defined categorically in 5-year age bins), female sex, dual-eligibility for Medicaid, share of months receiving the Part D low-income subsidy, original reason for entering Medicare being disability, Elixhauser comorbidities, physician fixed effects, and year. For race and ethnicity, White non-Hispanic is the reference category. Using marginal standardization, the adjusted value for White was 10.9 (95% CI 10.8 to 11.0). Errors are clustered at the physician level. 95% confidence interval in parentheses.

© 2021 Ly DP. *JAMA Health Forum.*

eTable 4: Racial and Ethnic Differences within Physician in Opioid Prescribing for Patients with New Low Back Pain (logistic model), 2007-2014

	Prescribing of any opioid during a given episode (odds ratio)
Race and ethnicity	
White, non-Hispanic	-
Black	0.85 (0.79 to 0.92)
Asian/Pacific Islander	0.71 (0.63 to 0.80)
Hispanic	0.91 (0.84 to 0.99)

Notes: Author’s calculation using Medicare data from 2006 to 2015. The examined time period is 2007-2014; 2006 data are used to look for a diagnosis code for low back pain or for a filled opioid prescription in the 365 days prior to a low back pain diagnosis in 2007, while 2015 data are used to look for filled opioid or NSAID prescriptions in the 365 days following a new diagnosis of low back pain in 2014. The multivariable logistic regression also controlled for age (defined categorically in 5-year age bins), female sex, dual-eligibility for Medicaid, share of months receiving the Part D low-income subsidy, original reason for entering Medicare being disability, Elixhauser comorbidities, physician fixed effects, and year. For race and ethnicity, White non-Hispanic is the reference category. 95% confidence interval in parentheses.

© 2021 Ly DP. *JAMA Health Forum.*

eTable 5: Racial and Ethnic Differences within Physician in Timing of Opioid Prescribing for Patients with New Low Back Pain, 2007-2014

	Prescribing of any opioid within 1 month of diagnosis (percentage points)	Prescribing of any opioid within 2 month of diagnosis (percentage points)	Prescribing of any opioid within 3 month of diagnosis (percentage points)
Race and ethnicity			
White, non-Hispanic	-	-	-
Black	-1.2 (-1.6 to -0.8)	-1.1 (-1.6 to -0.7)	-1.1 (-1.6 to -0.6)
Asian/Pacific Islander	-0.9 (-1.4 to -0.4)	-1.2 (-1.8 to -0.6)	-1.2 (-1.8 to -0.5)
Hispanic	-0.4 (-0.8 to 0.05)	-0.4 (-0.9 to 0.06)	-0.3 (-0.8 to 0.2)

Notes: Author's calculation using Medicare data from 2006 to 2015. The examined time period is 2007-2014; 2006 data are used to look for a diagnosis code for low back pain or for a filled opioid prescription in the 365 days prior to a low back pain diagnosis in 2007, while 2015 data are used to look for filled opioid or NSAID prescriptions in the 365 days following a new diagnosis of low back pain in 2014. The multivariable linear regression underlying these results included age (defined categorically in 5-year age bins), female sex, dual-eligibility for Medicaid, share of months receiving the Part D low-income subsidy, original reason for entering Medicare being disability, Elixhauser comorbidities, physician fixed effects, and year. For race and ethnicity, White non-Hispanic is the reference category. Using marginal standardization, the adjusted value for White for opioid prescribing within 1 month was 4.2 (95% CI 4.1 to 4.2), the adjusted value for White for opioid prescribing within 2 months was 5.3 (95% CI 5.2 to 5.3), and the adjusted value for White for opioid prescribing within 3 months was 6.0 (95% CI 6.0 to 6.1). Errors are clustered at the physician level. 95% confidence interval in parentheses.

© 2021 Ly DP. *JAMA Health Forum.*

eTable 6: Racial and Ethnic Differences within Physician in Opioid Prescribing and in Prescription NSAID Prescribing for Patients with New Low Back Pain, 2007-2014 (excluding those with any prescription NSAID use within 30 days or within 365 days prior to new low back pain diagnosis)

Panel A: Prescribing of Any Opioid

	Prescribing of any opioid during a given episode (percentage points), excluding those with prescription NSAID use in prior 30 days	Prescribing of any opioid during a given episode (percentage points), excluding those with prescription NSAID use in prior 365 days
Race and ethnicity		
White, non-Hispanic	-	-
Black	-1.5 (-2.3 to -0.8)	-1.7 (-2.6 to -0.8)
Asian/Pacific Islander	-2.7 (-3.6 to -1.8)	-2.9 (-4.0 to -1.9)
Hispanic	-1.3 (-2.1 to -0.5)	-1.8 (-2.7 to -0.9)

Panel B: Prescribing of Any Prescription NSAID

	Prescribing of any prescription NSAID during a given episode (percentage points), excluding those with prescription NSAID use in prior 30 days	Prescribing of any prescription NSAID during a given episode (percentage points), excluding those with prescription NSAID use in prior 365 days
Race and ethnicity		
White, non-Hispanic	-	-
Black	1.3 (0.4 to 2.2)	0.8 (-0.1 to 1.7)
Asian/Pacific Islander	2.1 (0.8 to 3.4)	2.7 (1.4 to 4.0)
Hispanic	2.9 (1.9 to 3.8)	1.9 (0.9 to 2.9)

Notes: Author's calculation using Medicare data from 2006 to 2015. The examined time period is 2007-2014; 2006 data are used to look for a diagnosis code for low back pain or for a filled opioid or NSAID prescription in the 365 days prior to a low back pain diagnosis in 2007, while 2015 data are used to look for filled opioid or NSAID prescriptions in the 365 days following a new diagnosis of low back pain in 2014. The multivariable linear regression underlying these results included age (defined categorically in 5-year age bins), female sex, dual-eligibility for Medicaid, share of months receiving the Part D low-income subsidy, original reason for entering Medicare being disability, Elixhauser comorbidities, physician fixed effects, and year. For race and ethnicity, White non-Hispanic is the reference category. Standard errors were clustered at the physician level. 95% confidence interval in parentheses.

© 2021 Ly DP. *JAMA Health Forum.*

eTable 7: Percent of Overall Racial and Ethnic Difference in Opioid Prescribing for Patients with New Low Back Pain Attributable to Within-Physician Differences, 2007-2014

	Prescription of any opioid
Within-physician difference	-0.0154
Between-physician difference	-0.0104
Percent of difference attributable to within-physician differences	59.6%

Notes: Author's calculation using Medicare data from 2006 to 2015. The examined time period is 2007-2014; 2006 data are used to look for a diagnosis code for low back pain or for a filled opioid prescription in the 365 days prior to a low back pain diagnosis in 2007, while 2015 data are used to look for filled opioid or NSAID prescriptions in the 365 days following a new diagnosis of low back pain in 2014. Details on the calculation are noted in the eMethods of this Supplement.

© 2021 Ly DP. *JAMA Health Forum.*
